# Screening and Identification of Target Gene of *StTCP7* Transcription Factor in Potato

**DOI:** 10.3390/ijms251910450

**Published:** 2024-09-27

**Authors:** Xingru Si, Wenjin Xu, Junliang Fan, Kaitong Wang, Ning Zhang, Huaijun Si

**Affiliations:** 1 State Key Laboratory of Aridland Crop Science, Gansu Agricultural University, Lanzhou 730070, China; 15129121974@163.com (X.S.); 19991938042@163.com (W.X.); fanjl@st.gsau.edu.cn (J.F.); kait_wang@163.com (K.W.); ningzh@gsau.edu.cn (N.Z.); 2 College of Life Science and Technology, Gansu Agricultural University, Lanzhou 730070, China; 3 College of Agronomy, Gansu Agricultural University, Lanzhou 730070, China

**Keywords:** potato, *StTCP7*, *StDAM5*, interaction

## Abstract

TCP transcription factors are involved in the regulation of plant growth and development and response to stress. Previous studies showed that *StTCP7* was involved in the abiotic stress response of potato and positively regulated plant tolerance to drought stress. On the basis of previous studies, this study verified the downstream target genes of *StTCP7* transcription factor binding through yeast one hybridization, double luciferase and other technologies, and conducted a preliminary analysis of the downstream target genes. The results showed that the *StTCP7* transcription factor could bind the promoter region of *StDAM5* and *StGOLS2* and regulate the expression of their genes. qRT-PCR analysis showed that the expression level of *StDAM5* gene was the highest in flower stalk tissue and the lowest in leaf stalk. The expression of *StGOLS2* gene was the highest in stem, the second in stalk, and the lower in root. Both *StDAM5* and *StGOLS2* genes responded to abiotic stress treated with 200 mM NaCl, 20% PEG-6000 and 100 µM ABA. The expression levels of target genes *StDAM5* and *StGOLS2* were up-regulated in *StTCP7* interfered plants. The protein encoded by the target gene *StDAM5* belongs to the Type II MADS-box protein, which contains 238 amino acids and is an acidic hydrophilic protein. The analysis of *StDAM5* promoter region showed that the promoter region of *StDAM5* gene contained cis-acting elements such as light response and abscisic acid. Subcellular localization showed that StDAM5 protein was expressed in both nucleus and cytoplasm.

## 1. Introduction

The potato (*Solanum tuberosum* L.), one of the world’s most important food crops, belongs to the Nightshade family [1]. The crop is known for its short growth cycle and mainly uses asexual reproduction. At the same time, in response to abiotic stress, potato growth can be regulated by a variety of genes or transcription factors through hormone signal induction, thus affecting the plant growth and development process.

As a DNA sequence located at the front end of gene coding region, promoter contains many cis-acting elements, which can bind to proteins specifically to achieve the regulation of gene transcription. Promoters and enhancers also regulate gene expression [2]. Therefore, the study of promoter is very important for plant growth and development.

Transcription factors (TFs), also known as trans-acting factors, bind specifically to homeopathic elements to ensure that the protein molecule is expressed by the target gene at a specific time with intensity and space. These include common TCP (Teosinte branched, Cycloidea, Proliferating cell factor) and MADS-box transcription factors. TCP was first reported in 1999 and named the TCP family after the first feature member [3]. An alkaline helix-ring-helix structure (bHLH domain) consisting of 59 amino acid residues [4] is divided into two categories: They are Class I (PCF or TCP-P) and Class Ⅱ (TCP-C), where the binding motif of class I is GGNCCCAC and the binding motif of class Ⅱ is GTGGNCCC [5]. MADS-box transcription factor, originally derived from cerevisiae transcription factor MINICHROMOSOME MAINTENANCE 1 (MCM1) [6], Arabidopsis Flower Homologous gene AGAMOUS (AG) [7], Goldfish Flower Homologous Gene of DEFICIENS (DEF) [8], and the initials of four members of the MADS-box gene family, Human SERUM RESPONSE FACTOR (SRF) [9]. The proteins encoded by the MADS-box family of genes contain about 58 conserved amino acid motifs at the n-terminal, called MADS-box domains, which bind specifically to DNA. MADS-box proteins are mainly divided into two types, namely type I and type II.

TCP transcription factors form autodimers or heterodimers that regulate downstream gene expression. For example, in *Arabidopsis thaliana*, *AtTCP15* and *AtTCP3* regulate the same target gene, *AtTCP15* regulates the expression of specific genes by affecting the auxin pathway, partially overlaps with the pathway regulating CIN II TCP protein, and binds to the promoter region of *SHY2* and *At1g29460*. These genes may be direct targets of TCP transcription factors [10]. TCP transcription factors have also been shown to be involved in regulating abiotic stress processes in plants in several species. For example, in rice (*Oryza sativa* L.) *Osa-miR319b* overexpression lines, the expression of its target genes *OsPCF6* and *OsTCP21* decreased, resulting in enhanced plant tolerance to cold stress [11]. Brassica pekinensis (*Brassica rapa* L.) *BraTCP4* can activate gene expression by binding to promoters of *WRKY75, WRKY70* and *WRKY33* to control plant stem rot resistance [12]. In rice, the expression of *OsNHX1* gene is induced by salt, drought and Abscisic acid ( ABA ) stress [13]. In maize (*Zea mays* L.), overexpression of *ZmTCP42* enhanced ABA hypersensitivity in seed germination and drought stress tolerance, thereby positively regulating plant tolerance to drought stress [14]. Liu et al. [15] showed that *MdTCP46* in apple (*Malus pumila* L.) inhibited the transcriptional activity of *MdABI5*, thereby negatively regulating the MdABI5-mediated ABA pathway in response to plant drought tolerance. Overexpression of *MdABI5* can activate the expression of *MdEM6* and *MdRD29A*, thereby increasing the sensitivity of plants to ABA signals and enhancing their drought resistance.

MADS-box transcription factors are also involved in important physiological processes such as growth, development, metabolism and abiotic stress of various plants [16]. It has been found that overexpression of *SlMBP11* in the MADS-box family in tomato (*Solanum lycopersicum* L.) may enhance tolerance to salt stress through ABA-independent signal transduction networks [17]. In maize, *ZmMADS9* and *ZmMADS10* respond to drought and cold stress [18]. In wheat (*Triticum aestivum* L.), overexpression of *TaMADS32* allogenic transformation of Arabidopsis thaliana enhances plant tolerance to abiotic stress [19]. In *Ginkgo biloba*, *GbMADS9* is up-regulated by plant hormones gibberellic acid (GA_3_) and ABA in response to salt, drought and cold stress [20].

Studies have shown that tomato *SlTCP7* is involved in morphogenesis and response to abiotic stress during crop growth and development [21], while potato *StTCP7* is involved in regulating plant adaptability to drought stress [22] through overexpression and inhibitory expression. In this study, the downstream target genes regulated by *StTCP7* in ABA signaling pathway were verified by yeast single hybridization technique and dual luciferase complementary analysis. qRT-PCR (Real-time quantitative PCR) was used to verify the expression changes of target genes in abiotic stress, different tissues of potato and *StTCP7* interfered plants, and the target gene *StDAM5* was preliminarily analyzed. It provides a theoretical basis for further study on *StTCP7* gene co-regulating plant growth and development with other genes.

## 2. Results

### 2.1. Analysis of Promoter Elements of Target Genes

**Figure 1:**
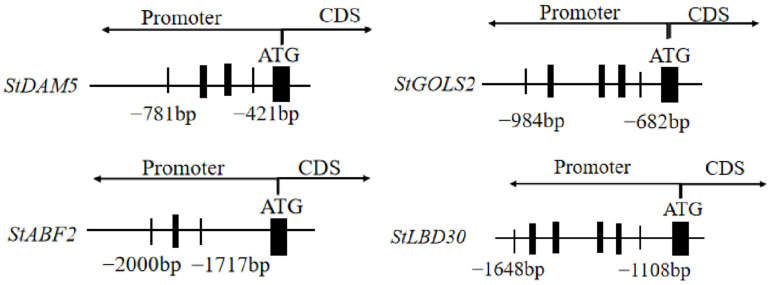
Sequence analysis of *StTCP7* target gene promoter. 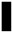 Binding motif; 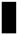 ATG; 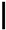 Selected truncation sequence.

*Cis*-acting elements were analyzed for 2000 bp upstream of target genes *StDAM5*, *StABF2*, *StGOLS2* and *StLBD30*, and it was found that all of them contained multiple abiotic stress-related elements and binding motifs with varying amounts. The promoter region of *StDAM5*, *StABF2*, *StGOLS2* and *StLBD30* contains 2, 1, 3 and 4 combined core acting elements, respectively (Figure 1).

### 2.2. Self-Activation Detection of Bait Vector

**Figure 2:**
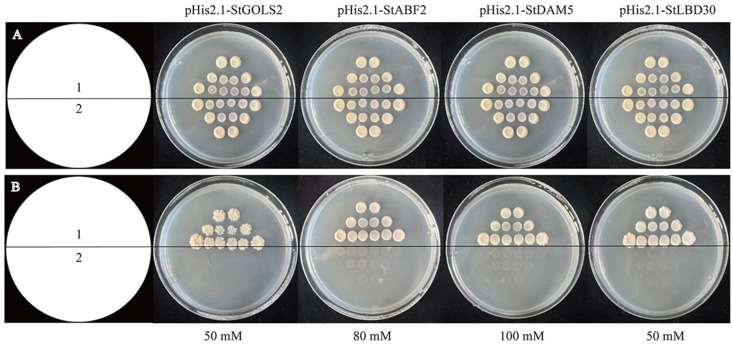
Self-activation assay for bait vector. (**A**): Medium of SD/−Trp/−His; (**B**): Medium of SD/−Trp/−His+3-AT; 1: Vector of p53-His2.1; 2: Bait vector.

The electrophoresis results of RCR products of *StTCP7* and its target genes *StGOLS2*, *StABF2*, *StDAM5* and *StLBD30* are shown in Figures S1 and S2. The obtained positive strain was transferred into Y187 yeast cells containing p53-His2.1 as positive control, and then applied to 3-AT SD/−Trp/−His defective solid medium containing different concentration gradients. The results showed that the 3-AT concentrations that could inhibit the leakage expression of His3 protein on each target gene vector were 50 mM, 80 mM, 100 mM and 50 mM, respectively (Figure 2).

### 2.3. Yeast One Hybridization Verification

**Figure 3:**
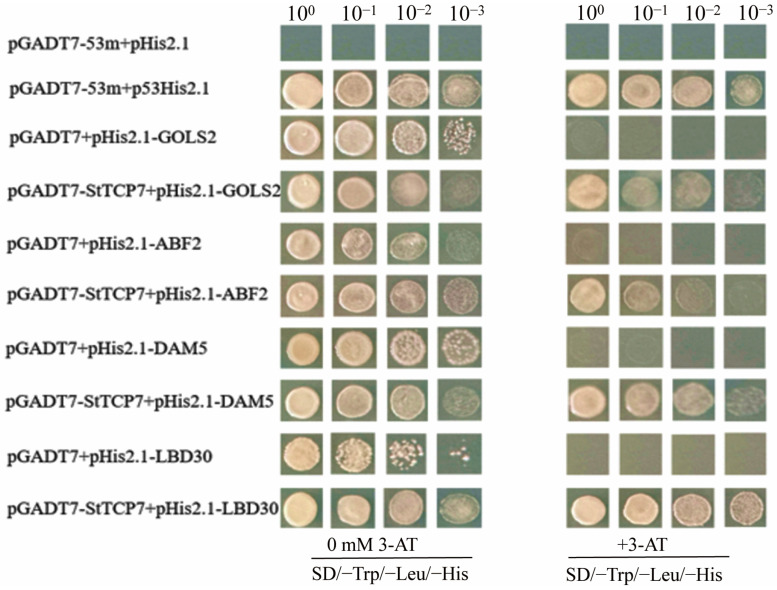
Analysis of yeast one hybridization interaction.

The experimental group, positive control and negative control were coated on SD/−Trp/−Leu di-deficiency solid defect medium, and single colonies were diluted with ddH_2_O to 10^−3^, 10^−2^, 10^−1^ and 10^0^ times, respectively. Then, they were separately coated in SD/-Trp/-Leu/-His triple-deficiency solid defect medium containing corresponding inhibitory 3-AT concentration, and placed in a constant temperature incubator at 29 °C for 48–96 h. It was found that positive control grew normally, while negative control did not grow. Yeast cells of different concentrations in the experimental group could grow in the 3-deficient solid medium of 0 mM 3-AT and SD/−Trp/−Leu/−His containing corresponding inhibitory concentration of 3-AT (Figure 3). These results indicated that potato *StTCP7* could bind to the promoter region of *StDAM5*, *StABF2*, *StGOLS2* and *StLBD30* genes to initiate the expression of downstream reporter gene His3, thus preliminarily confirming that *StTCP7* gene might regulate the expression of the above four genes.

### 2.4. Verification by Double Luciferase Test

**Figure 4:**
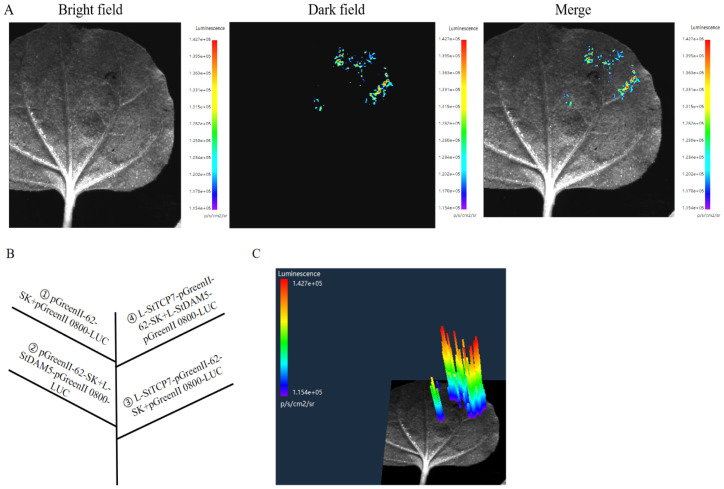
Double luciferase assay verified *StDAM5* interaction analysis. (**A**): The observation of fluorescent; (**B**): The injection position of different combinations: (**C**): Fluorescence intensity. The control combination was ① pGreenII-62-SK (Empty vector) + pGreenII 0800-LUC (Empty vector); ② pGreenII-62-SK+L-StDAM5-pGreenII 0800-LUC; ③ L-StTCP7-pGreenII-62-SK+pGreenII 0800-LUC. The experimental combination is ④ L-StTCP7-pGreenII-62-SK+L-StDAM5-pGreenII 0800-LUC.

**Figure 5:**
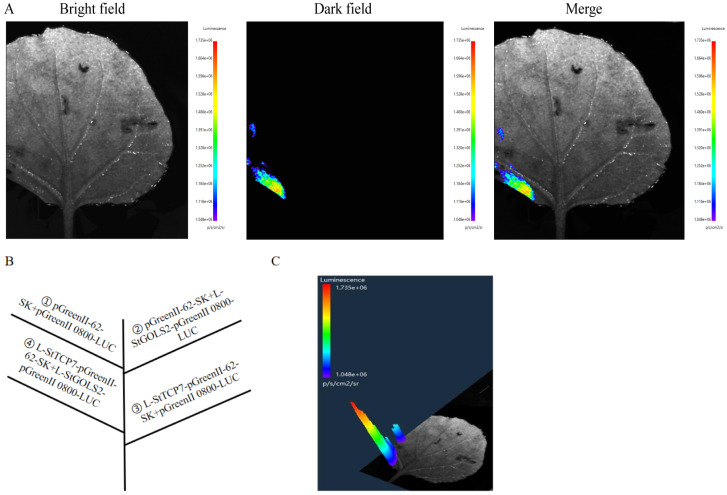
Double luciferase assay verified *StGOLS2* interaction analysis. (**A**): The observation of fluorescent; (**B**): The injection position of different combinations: (**C**): Fluorescence intensity. The control combination was ① pGreenII-62-SK (Empty vector) + pGreenII 0800-LUC (Empty vector); ② pGreenII-62-SK+L-StGOLS2-pGreenII 0800-LUC; ③ L-StTCP7-pGreenII-62-SK+pGreenII 0800-LUC. The experimental combination is ④ L-StTCP7-pGreenII-62-SK+L-StGOLS2-pGreenII 0800-LUC.

In order to further analyze the effect of *StTCP7* transcription factor on the transcriptional activity of the promoter regions of *StDAM5*, *StABF2*, *StGOLS2* and *StLBD30* genes, the dual luciferase reporter gene detection system was used to verify the regulatory relationship in tobacco (*Nicotiana tabacum* L.) leaves. The results showed that only the combination of *StTCP7*, *StDAM5* and *StGOLS2* could observe fluorescence signals in tobacco, while the combination of *StTCP7*, *StABF2* and *StLBD30* could not detect fluorescence signals (Figure 4 and Figure 5). The above tests showed that the combination of *StTCP7*, *StABF2* and *StLBD30* gene promoter region did not detect fluorescence signals, which was inconsistent with the results of the previous yeast monohybrid analysis, and it was speculated that it might be due to the high false positives in yeast, which needs further verification. *StTCP7* can bind to the promoter region of *StDAM5* and *StGOLS2* and regulate the expression of their genes, which is consistent with the results of yeast one hybridization verification.

**Figure 6:**
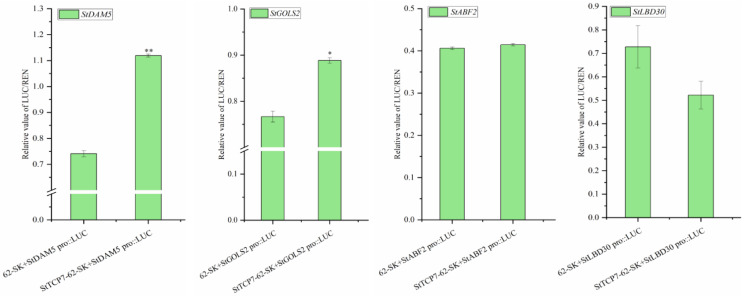
Relative activity value of LUC (Firefly luciferase LUC/Sea kidney luciferase REN). A one-way ANOVA was used in this experiment and the error bars represent the standard errors (*n* = 3). Asterisks indicate significant differences: *, *p* < 0.05; **, *p* < 0.01.

The co-transposed luciferase effector vector and reporter vector were injected into tobacco leaves, and the ratio of LUC and REN in tobacco leaves was determined to analyze the promoter activity. There was no significant difference between StTCP7-62-SK+StABF2 *pro*::LUC and experimental group StTCP7-62-SK+StLBD30 *pro*::LUC (Figure 6). There is no regulatory relationship between StTCP7 transcription factor, *StABF2* and *StLBD30*. The difference between StTCP7-62-SK +StDAM5 *pro*::LUC test group and control group was very significant, indicating that *StTCP7* transcription factor can bind the *StDAM5* promoter region and regulate its gene expression. The difference between StTCP7-62-SK +StGOLS2 *pro*::LUC test group and control group was significant, indicating that *StTCP7* transcription factor can bind to *StGOLS2* promoter region and regulate its gene expression.

### 2.5. qRT-PCR Analysis

**Figure 7:**
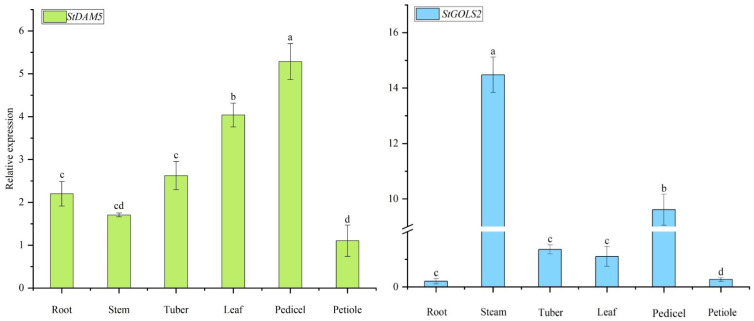
The analysis of tissue-specific expression of downstream target genes in the potato variety ‘Atlantic’. A one-way ANOVA was used in this experiment and the error bars represent the standard errors (*n* = 3). Different small letters mean significant differences (*p* < 0.05).

The expression patterns of *StDAM5* and *StGOLS2* downstream target genes of *StTCP7* transcription factor in different tissues of potato variety ‘Atlantic’ were analyzed by qRT-PCR.As shown in Figure 7, the expression level of *StDAM5* gene was the highest in stalk tissue and the lowest in petiole. The expression of *StGOLS2* gene was the highest in stem, the second in stalk, and the lowest in root.

**Figure 8:**
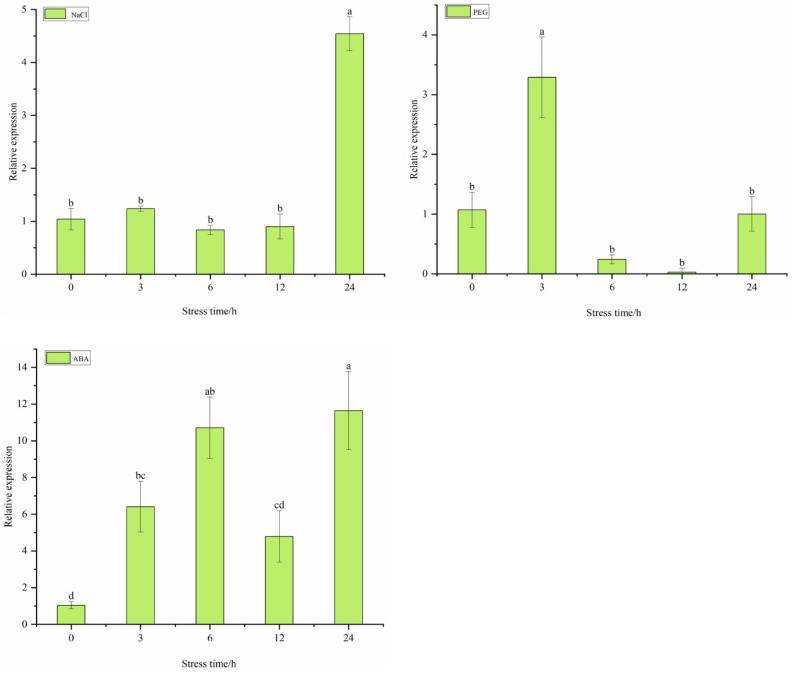
Changes in *StDAM5* gene expression under abiotic stress conditions. Significant differences in relative gene expression between different stress times in the same tissue (*p* < 0.05, *n* = 3). The concentrations in the graph are NaCl: 200 mM NaCl; PEG: 20% PEG-6000; ABA: 100 µM ABA.

To determine whether target genes *StDAM5* and *StGOLS2* respond to abiotic stress of salt, drought and ABA pathway, potato variety ‘Atlantic’ was treated with NaCl, Polyethylene glycol ( PEG-6000) and ABA. NaCl stress is a very specific. In a high-salt environment, a large amount of Na^+^ accumulated in plant cytoplasm will break the Na^+^ /K^+^ balance of cells, reduce the ability of plant cells to maintain the dynamic balance of internal and external ions, and then hinder the absorption of other ions (such as K^+^), and ultimately affect the primary and secondary metabolism of plants. The results showed that the target gene *StDAM5* was generally up-regulated under NaCl treatment, and the expression reached the maximum after 24 h of NaCl treatment. The expression of 20% PEG-6000 was down-regulated, and the expression level was the lowest at 12 h. Under ABA treatment, its expression levels showed a trend of first increasing, then decreasing and then increasing, and the overall expression was upregulated (Figure 8).

**Figure 9:**
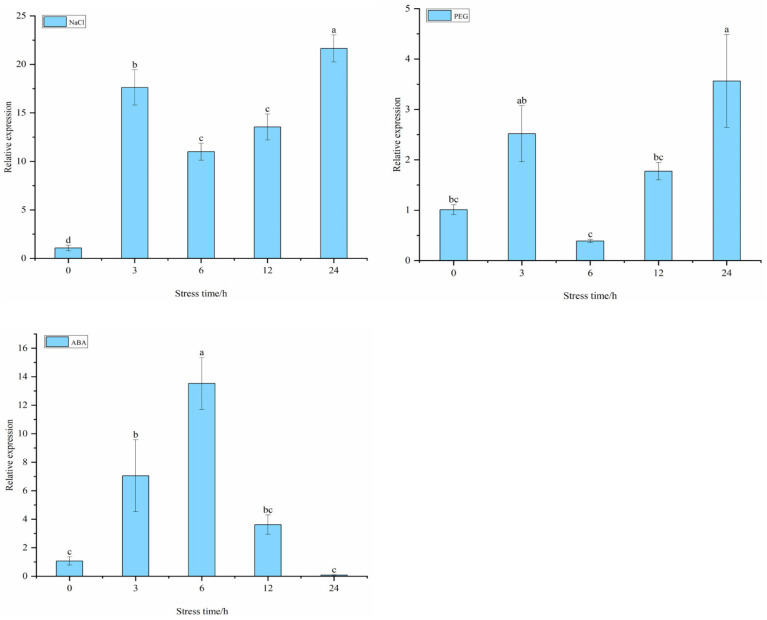
Changes of *StGOLS2* gene expression under abiotic stress. Significant differences in relative gene expression between different stress times in the same tissue (*p* < 0.05, *n* = 3). The concentrations in the graph are NaCl: 200 mM NaCl; PEG: 20% PEG-6000; ABA: 100 µM ABA.

The expression of target gene *StGOLS2* was generally upregulated under NaCl treatment, upregulated under PEG-6000 treatment, and initially increased and then decreased under ABA treatment, showing overall down-regulated expression (Figure 9). This suggests that potato *StDAM5* and *StGOLS2* genes may respond to abiotic stress of salt, drought and ABA pathway.

**Figure 10:**
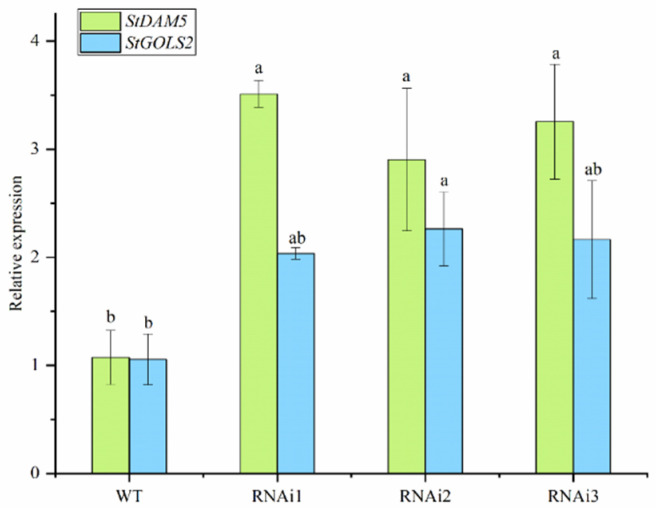
Relative expression level of target gene in *StTCP7* in transgenic plants compared with WT. Different small letters mean significant differences *(p* < 0.05, *n* = 3).

To further verify the above interaction analysis results, qRT-PCR was used to verify the relative expression levels of target genes *StDAM5* and *StGOLS2* in *StTCP7* interference expression plants (RNAi). The results showed that compared with wild type (WT), the expression levels of target genes *StDAM5* and *StGOLS2* were significantly increased (Figure 10) *StTCP7* down-regulated the expression of *StDAM5* and *StGOLS2* genes.

### 2.6. Bioinformatics Analysis of Target Gene StDAM5

The gene was obtained from the NCBI website, showing that it was located on potato chromosome 11, with a total length of 5945 bp and a CDS region length of 717 bp, belonging to the broken gene and containing 7 introns (Figure S3).

The physical and chemical properties of *StDAM5* protein showed that it contained 238 amino acids, the relative molecular weight was 27 kD, the theoretical isoelectric point was 5.50, and the chemical formula was C_1158_H_1900_N_344_O_388_S_10_, that is, it was an acidic protein.

Among the 19 amino acids of *StDAM5* protein, there were 34 positively charged amino acid residues Arg and Lys, and 38 negatively charged amino acid residues Asp and Glu. The instability index is 56.90, with a value greater than 40, indicating that this protein is unstable, the fat coefficient is 79.03, and the total mean hydrophilicity is −0.873.

Transmembrane domain prediction shows that potato *StDAM5* protein has no transmembrane region and no transmembrane domain, and belongs to non-transmembrane and hydrophilic protein (Figure S4).

The prediction of secondary structure and tertiary structure showed that the gene contained 76 amino acids with random curl, accounting for 31.93%. α-helix contains 130 amino acids, accounting for 54.62%; The extended chain contains 26 amino acids, accounting for 10.92%. β-corner contains 6 amino acids, accounting for 2.52% (Figures S5 and S6).

In order to further study the physiological functions involved in potato *StDAM5* gene, cis-acting elements were analyzed on the 2 kb upstream sequence of *StDAM5* gene initiation codon ATG. The results showed that the upstream promoter region of the gene contained photo responsive elements, abscisic acid, anaerobic induction and other related cis-acting elements (Table 1).

Signal peptide analysis was performed on potato *StDAM5* protein (Figure S7), indicating no signal peptide. By analyzing the functional domain of *StDAM5*, it was found that *StDAM5* contains two domains, namely MADS at positions 1–60 and K-box conserved domain at positions 87–172, which belong to the MADS-box protein of Type II (Figure S8).

Through BLAST search, a total of 17 species of amino acid sequences with high homology to potato *StDAM5* protein were obtained, including tomato, tobacco, pannali tomatoes (*Solanum pennellii* L.), pepper(*Capsicum annuum* L.), *Solanum dulcamara* and wolfberry (*Lycium ferocissimum* L.). The similarity between *StDAM5* protein and WT potato (*Solanum verrucosum* L.) was 97.92%. The similarity to yellow carrot was the lowest, 78.05%. The phylogenetic tree results showed that potato *StDAM5* was the closest relative to WT potato and the furthest relative to yellow carrot on the evolutionary level (Figures S9 and S10).

### 2.7. Subcellular Localization of the Target Gene StDAM5

**Figure 11:**
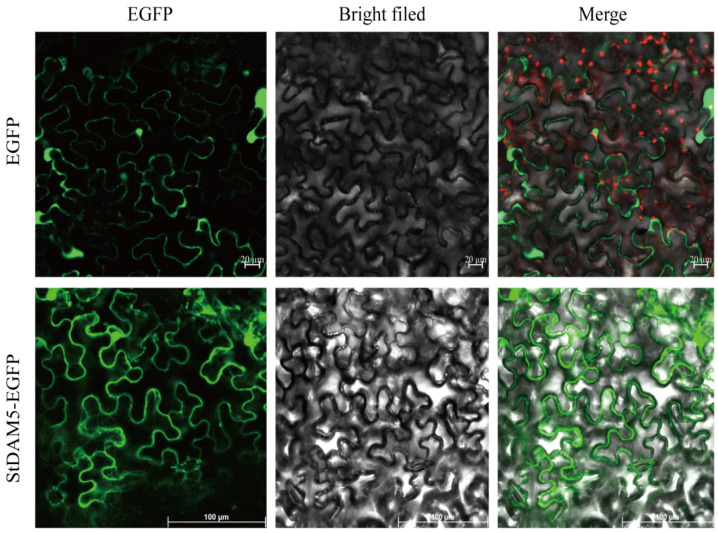
Subcellular localization of pEGFP-StDAM5. EGFP: Blank control (EGFP fluorescence signal in the dark field); StDAM5-EGFP:Fusion protein of StDAM5 and EGFP(Cell morphology under bright field); Merged: Combination field; bar = 20 μm,100 μm.

The StDAM5-EGFP recombinant fusion protein was observed using the EGFP empty vector as a blank control. The results showed that the recombinant fusion protein had EGFP signals in both nucleus and cytoplasm of tobacco leaves, indicating that potato StDAM5 protein was expressed in both nucleus and cytoplasm (Figure 11).

## 3. Discussion

Due to the influence of the surrounding environmental factors, plant growth and development have evolved a variety of regulatory mechanisms to respond to the balance between growth and development and stress, among which the TCP transcription factor family also plays an indispensable role [13,23,24]. Existing studies have shown that miR319 targets the TCP gene family and encodes plant-specific transcription factors, which in turn regulate plant tolerance to abiotic stresses. Of course, TCP transcription factors can also regulate other stress tolerance genes through hormone signaling pathways, thus affecting plant growth and developmental processes.

The potato *StTCP7* gene belongs to the PCF class subfamily with the binding motif GGNCCCAC [5]. In this experiment, the sequence results of the first 2000 bp of the promoter regions of the potential downstream target genes *StDAM5, StABF2, StGOLS2,* and *StLBD30* were analyzed, and it was found that all of them contained multiple abiotic stress-related elements and their varying numbers of binding motifs, containing two, one, three, and four binding core elements, respectively. Therefore, it is hypothesized that the potato *StTCP7* transcription factor may regulate its gene expression by binding to the promoter regions of the above four genes.

To verify the above speculation, this experiment was analyzed by yeast single hybridization technique and dual luciferase analysis. The results showed that *StTCP7* could bind *StDAM5* and *StGOLS2* promoter regions and regulate their gene expression. Afterwards, the reliability of the test results was further verified from the quantitative point of view, and the results were consistent with the previous conclusions. It has been shown that in rice, *OsWRKY63* positively regulates the *OsDREB1* resistance pathway as verified by yeast single heterozygote and luciferase assays, and rice cold tolerance is negatively regulated through the *OsWRKY63-OsWRKY76-OsDREB1B* transcriptional cascade [25]. In cotton (*Gossypium hirsutum* L.), *GbTCP5* can affect the transcript levels of *GbGLOS2*, *GbUBC19*, and *GbERD7* genes attenuating drought tolerance in cotton [26]. In peach (*Prunus persica* L.) [27], *PpTCP20* was found to repress the expression of *PpDAM5* and *PpDAM6* in the MADS-box family by luciferase assay. These findings are similar to those of the present experimental study, suggesting that the *StTCP7* transcription factor may regulate potato growth and development by binding to the promoter regions of the downstream target genes *StDAM5* and *StGOLS2*.

The expression patterns of target genes *StDAM5* and *StGOLS2* in different potato tissues were analyzed by qRT-PCR. The results showed that the expression level of *StDAM5* was the highest in stalk tissues. The expression of *StGOLS2* gene was the highest in stems. The potato variety ‘Atlantic’ was treated with different concentrations of NaCl, PEG-6000 and ABA, and the relative expression levels of RNAi in *StTCP7* interfered plants were analyzed. The results showed that the target gene *StDAM5* was generally up-regulated under salt stress and ABA treatment, and down-regulated under PEG-6000 treatment. The target gene *StGOLS2* was generally up-regulated under NaCl and PEG-6000 treatment, and down-regulated under ABA treatment. Compared with WT, the expression levels of target genes *StDAM5* and *StGOLS2* in RNAi of *StTCP7* interfered plants were significantly increased. In conclusion, *StTCP7* transcription factor may affect the expression of *StDAM5* and *StGOLS2* genes through hormone signal transduction, thereby responding to abiotic stress tolerance in potato plants during growth and development, which is consistent with the research results of Almeida et al. [5,28]. Studies have shown that TCP transcription factors are involved in many regulatory mechanisms of plant hormone response to abiotic stress through interaction with other genes. For example, in grapes (*Vitis vinifera* L.), MADS-box transcription factors *VyAGL42* and *VyMADS23* can synergistically enhance drought resistance of grapes by directly binding to the promoter of *VyP5CR* gene [29]. In White birch (*Betula platyphylla* L.), *BpTCP7* was found to positively regulate salt and drought stress responses after being treated with NaCl and PEG [30]. In rice, *OsPCF6* and *OsTCP21* enhance cold stress tolerance by changing the scavenging capacity of reactive oxygen species, thus affecting plant growth and development [11]. In apples, *MdTCP46* inhibits *MdABI5* transcriptional activity, thereby negatively affecting MDABI5-mediated ABA signaling and drought response [15]. In summary, the subsequent regulation of *StDAM5* and *StGOLS2* expression by *StTCP7* transcription factor in response to hormone signaling pathway, and thus affecting plant tolerance to abiotic stress, requires further verification and exploration.

*StTCP7* transcription factor can regulate the expression of downstream target genes, so the downstream target gene *StDAM5* was used as the entry point for bioinformatics analysis and subcellular localization. In order to provide a theoretical reference for the transcriptional cascade regulation of TCP and MADS-box on plant growth and development and resistance to abiotic stress.

Bioinformatics studies showed that *StDAM5* gene belonged to the MADS-box protein of Type II, located on chromosome 11 of potato, and was a broken gene with 7 introns. The physicochemical properties of the protein showed that the protein was acidic protein. This protein is unstable and belongs to hydrophilic protein. In terms of evolutionary degree, it is most closely related to WT potato and most closely related to yellow carrot. Analysis of cis-acting elements shows that it contains related cis-acting elements such as photo response, abscisic acid, and anaerobic induction, indicating that it may participate in the signal-induced stress process [31,32,33,34]. Recent studies have shown that this transcription factor regulates plant stress tolerance through exogenous hormone treatment or in coordination with other genes. For example, in trifoliate orange (*Citrus sinensis* L.), the MADS-box family gene *PtrANR1* can activate its transcriptional expression by binding to GArG-box in the promoter of *PtrAUX1*, thereby increasing IAA content and promoting root growth and drought resistance of plants [35]. In Arabidopsis thaliana, the MADS-Box transcription factor *AGL16* binds to *CYP707A3, AAO3*, and *SDD1* promoters and regulates their transcription, resulting in altered ABA levels and acting as a negative regulator of drought resistance [36]. In cotton, MADS-box transcription factor *GhFYF* interacts with HAD-like protein *GhGPP2* to participate in salt stress response in plants [37]. In conclusion, *StDAM5* gene in potato may be involved in the regulation of hormone levels, thus affecting the drought stress tolerance of potato. Therefore, it is possible to further study the abiotic stress processes involved in *StDAM5* gene from its hormone signaling pathway in future studies.

Subcellular localization showed that potato StDAM5 protein was localized in the nucleus and cytoplasm, which was similar to the results of MADS-box transcription factors localization shown in current studies, such as the four types of proteins PaMADS2, PaMADS4, PaMADS5 and PaMADS7 were localized in the nucleus [38]. In cotton, the APETALA1 (AP1) protein in the MADS-box gene family is localized in the nucleus and acts as a positive regulator of plant flowering [39]. In the peach genome, eight MADS-boxes and one AP2/EREBP protein are localized in the nucleus [40]. In Phalaenopsis lantao (*Phalaenopsis equestris* L.), subcellular localization and protein-protein interaction analysis revealed that PeMADS28 can form homodimers as well as heterodimers with class D and E MADS-box proteins, localized in the nucleus and cytoplasm [41]. The nucleus acts as the regulatory center of cellular genetics and metabolism, and the functional expression of genes is mainly regulated by proteins [42]. Thus, subcellular localization can provide a theoretical basis for its subsequent experiments by locating the position of the protein and thus determining the involvement of the *StDAM5* gene in the regulation of plant growth and developmental activities through the nucleus and cytoplasm.

## 4. Materials and Methods

### 4.1. Plant Materials and Growth Conditions

The test tube seedlings of potato variety ‘Atlantic’ were transplanted into POTS for soil culture. After 30 days of culture, selected plants with the same growth were treated with 20% PEG-6000, 200 mM NaCl and 100 µM ABA. The treatment methods were cultured under the condition of illumination intensity of 20,000 Lx, 16 h/d and temperature of (21 ± 2) °C, in which 200 mM NaCl and 20% PEG6000 were irrigated by solution, and 100 µM ABA was sprayed by leaf surface in hormone treatment. The sampling time was 0, 3, 6, 12, 24 h, the first **1**–**3** compound leaves at the top of the plant were randomly collected, and the sampling were repeated for 3 times in each group. After forming potatoes, the roots, petioles, stems, stolons, tubers, leaves and flower stalks are collected. Tobacco seeds were planted in a 10 cm × 10 cm pot with a 2:1 ratio of nutrient soil and vermiculite, and cultured under a light intensity of 20,000 Lx, 16 h/d, and temperature of (21 ± 2) °C for about 30 days.

Transgenic potato *StTCP7* seedlings were inoculated in MS medium containing 3% sucrose and subcultured under the conditions of temperature 22 °C, light period 16 h light/8 h darkness.

### 4.2. Analysis of Target Gene Promoter Elements of StTCP7 Transcription Factor

Based on literature search [26,27], four target genes that may be the transcription factor of potato *StTCP7* were obtained. *StDAM5* (Soltu.DM.11G003050.1), *StABF2* (Soltu.DM.04G033590.1), *StGOLS2* (Soltu.DM.01G0252302) and *StLBD30* (Soltu.DM.01G037670.1), respectively. The entry number was submitted to the NCBI and Spud DB Potato Genomics Resource websites for comparison to find the promoter sequence, and the combination sequence GGNCCCAC.

### 4.3. Construction of Yeast One Hybridization Vector

The sequence information of *StTCP7* (XM_006348485-2) gene was retrieved from NCBI website, and the CDS region of *StTCP7* gene was cloned using the leaf cDNA of potato variety ‘Atlantic’ as template. Design at the same time with the same arm and enzyme digestion site specific primers, the sequence is: StTCP7-F: CAGTGAATTCCACCCGGGATGTCGACGTCGGTAGAACC, StTCP7-R: TTCATCTGCAGCTCGAGCTCTCATTGTCCATCATCATCCCTTCTC, including enzyme loci for *Sma* I and *Sac* I. The reaction system was 10.0 μL Premix *Taq*™ (TaKaRa *Taq*™ Version 2.0 plus dye), 1.0 μL cDNA template, 1.0 μL StTCP7-F and StTCP7-R, and 7.0 μL ddH_2_O. The reaction conditions were predenaturation at 95 °C for 3 min, denaturation at 94 °C for 25 s, annealing at 60 °C for 25 s, extension at 72 °C for 1 min, a total of 34 cycles, final extension at 72 °C for 5 min, and insulation at 4 °C.

The PCR products were detected by 1% agarose gel electrophoresis and the target fragments were recovered (follow the instructions of the SanPrep column DNA glue recovery kit). The recombinant vector was constructed by homologous recombinant method, and the pGADT7 carrier plasmid was extracted and recovered after enzyme digestion. The enzyme digestion reaction system was as follows: 1.0 μL *Sma* I, 1.0 μL *Sac* I, 2.0 μL 10× Quick Cut Buffer, 8.0 μL pGADT7 carrier plasmid, 8.0 µL ddH_2_O with a total of 20 μL system. Recombinant reaction system 5 µL 2 ×Uniclone Seamless Cloning Mix, 2 µL linearized vector, 2 µL insertion fragment, 1.0 µL ddH_2_O system with a total of 10 µL. After that, it was introduced into Escherichia coli receptor cell DH5α, and the specific operation was referred to the method of Fangfang Wang [43], named pGADT7-StTCP7.

Amplification was performed according to the specific primers obtained in Section 2.2 (Table S1). The PCR products were detected by electrophoresis with 1% agarose gel, the target fragments were recovered, and the recombinant vectors were constructed by homologous recombination method, the method is the same as above.The recombinant bait vectors were named pHis2.1-StGOLS2, pHis2.1-StABF2, pHis2.1-StDAM5 and pHis2.1-StLBD30 respectively.

### 4.4. Self-Activation Detection of Bait Carrier

The bait vector plasmid was transformed into yeast receptive Y187 cells, ddH_2_O was suspended, and coated with SD/− Trp/− His defect medium containing 0 mM, 10 mM, 30 mM, 50 mM, 80 mM and 100 mM 3-AT, respectively. Then it was placed in a constant temperature incubator at 29 °C for 48–96 h, and the appropriate 3-AT concentration was screened.

### 4.5. Yeast One Hybridization Test

Bait vector and reporter vector pGADT7-StTCP7 plasmid were co-transferred to Y187 yeast cells as the experimental group, pGADT7-53m and p53His were co-transferred to Y187 yeast cells as the positive control, and pGADT7-53m and pHis2.1 co-transformed Y187 yeast cells as the negative control. The single colonies were diluted to 10^−3^, 10^−2^, 10^−1^ and 10^0^ times with ddH_2_O, and then coated on the defective solid medium containing SD/− Trp/− Leu/−His and SD/ − Trp/− Leu/− His/3-AT, respectively. After incubating at 30 °C for 48–96 h, the colony growth was observed.

### 4.6. Double Luciferase Test

pGreenII-0800-LUC was used as reporter gene vector. The upstream and downstream sequences of potential downstream target genes containing restriction sites were used as primers for PCR amplification and homologous recombination. The obtained fusion expression vector is used as the reporter gene. They are named L-StDAM5-pGreenII 0800-LUC, L-StABF2-pGreenII 0800-LUC, L-StGOLS2-pGreenII 0800-LUC, L-StLBD30-pGreenII 0800-LUC.

The full length of CDS region of *StTCP7* gene was amplified with specific primers containing restriction restriction sites *Sac* I and *Sma* I. L-upstream primer sequences respectively StTCP7-pGreenII-62-SK-F: ggacagcccaagctgagctcATGTCGACGTCGGTAGAACC, downstream L-StTCP7-pGreenII-62-SK-R: gaattcctgcagcccgggTCATTGTCCATCATCATCCCTTCTC. The resulting fusion expression vector was used as an effector and was named L-StTCP7-pGreenII-62-SK.

The reporter gene, effector factor and pGreenII-62-SK empty vectors were respectively introduced into agrobacterium tumefaciens GV3101 receptor cells by freeze-thaw method and mixed in the ratio of 1:10. The vector containing reporter gene was co-transferred with pGreenII-62-SK empty vector as the control group, and the vector containing effector factor was co-transferred with the vector containing reporter gene as the experimental group. After conversion, samples were collected 2–3 days later.

Finally, the LUC activity was normalized to REN activity, and the relative activity of LUC was calculated using the luciferase of sea kidney as the internal reference. All experiments were independently repeated three times.

### 4.7. qRT-PCR Analysis Method

The qRT-PCR specific primers were designed through the online website Preimer 3 Plus, and potato *StEF1α* gene (GenBank No. AB061263.1) was used as the internal reference gene (Table S2). The expression levels of downstream target genes of potato *StTCP7* under abiotic stress and in transgenic plants were analyzed. The reaction system and reaction conditions refer to Xiao Wang et al. [44]. The 2^−ΔΔCt^ method was used to calculate the relative expression of downstream target genes [45].

Extraction of RNA and synthesis of cDNA strands in potato were extracted according to the Trizol method, and reverse transcription was performed using the TIANGEN cDNA First Strand cDNA Synthesis Kit. The specific operation was carried out according to the product instructions.

### 4.8. Bioinformatics and Expression Analysis of Target Gene StDAM5

The upstream 2000 bp sequence of initiation codon of potato *StDAM5* gene was obtained by NCBI. The basic physicochemical properties, cis-acting elements and Gene Structure were analyzed by ProtParam, PlantCARE and Gene Structure Dispaly Server. Protein hydrophobicity, amino acid transmembrane structure analysis and protein signal peptide prediction were performed using online websites ProtScale, TMHMMServer v.2.0 and SignaIP. The online websites SOPMA, SWISS-MODEL and SMART: Main page were used to predict protein secondary and tertiary structures and protein conserved domains. The homologous amino acid sequences of 17 species including tomato were obtained by BLAST comparison with the amino acid sequences of *StDAM5*, and the protein sequences were analyzed by DNAMAN6.0 and MEGA7.0 software and the homologous protein evolutionary tree was constructed (Table S3).

### 4.9. Subcellular Localization of Target Gene StDAM5

*StDAM5* gene sequence (XM_00635313.2) was retrieved from potato Spud DB and primers were designed. Primer sequences for StDAM5 -F: CGGGGGACGAGCTCGGTACCATGGCTAGAGAAAAAATTCAGAT, StDAM5-R: TGCTCACCATGTCGACACCTGAGTAAGGTAGCCCCA, including enzyme loci for *Kpn* I and *Sal* I. The PCR amplification reaction system and reaction conditions were the same as those in Section 4.3. The amplified fragment was connected to the pCAMBIA1300-35S-EGFP vector by homologous recombination method, named StDAM5-EGFP.

pCAMBIA1300-35S-EGFP unloaded plasmid and StDAM5-EGFP plasmid were transformed into *Agrobacterium rhizoma* GV3101 receptor cells by freeze-thawed method, respectively. Agrobacterium solution containing Pcambia130-35S-EGFP no-load plasmid was used as the control group, and Agrobacterium solution containing StDAM5-EGFP recombinant vector was used as the experimental group.

## 5. Conclusions

*StTCP7* was cloned from the potato variety ‘Atlantic’. By using yeast one hybridization technique and double luciferase assay, it was found that *StTCP7* transcription factor binds to the promoter region of target genes *StDAM5* and *StGOLS2* with different activity levels, and regulates the expression of their genes. The expression analysis showed that *StDAM5* gene expression was the highest in stalk tissue and the lowest in petiole. The expression of *StGOLS2* gene was the highest in stem and the lowest in root. Under 200 mM NaCl, 20% PEG-6000 and 100 µM ABA stress, *StDAM5* and *StGOLS2* genes were responsive to abiotic stress. The expression levels of StDAM5 and *StGOLS2* were up-regulated in *StTCP7* interfered plants. *StDAM5* gene belongs to the MADS-box protein of Type II. In the degree of evolution, it is most closely related to WT potato and most distantly related to yellow carrot. The promoter region of this gene contains cis-acting elements such as light response and abscisic acid response. Subcellular localization indicated that StDAM5 protein was localized in the nucleus and cytoplasm. These results can provide a theoretical basis for further analysis of potato *StTCP7* gene and other genes co-regulation of plant growth and development.

## Figures and Tables

**Table 1:** Prediction of *cis*-acting elements of 2000 bp promoter upstream of *StDAM5*.

Name	Sequence	Account	Function
ABRE	ACGTG	3	*cis*-acting element involved in the abscisic acid responsiveness
AE-box	AGAAACAA/AGAAACTT	2	part of a module for light response
ARE	AAACCA	3	*cis*-acting regulatory element essential for the anaerobic induction
CAAT-box	CAAAT/CAAT/CCAAT/CCCAATTT	29	common *cis*-acting element in promoter and enhancer regions
G-Box	CACGTT	3	*cis*-acting regulatory element involved in light responsiveness
MYB	TAACCA	1	

## Data Availability

Data are contained within the article and Supplementary Materials.

## Supplementary Materials

The following supporting information can be downloaded at: https://www.mdpi.com/article/10.3390/ijms251910450/s1.
