# Supplementary material for: Screening and Identification of Target Gene of StTCP7 Transcription Factor in Potato
Source: Int J Mol Sci. 2024 Sep 27;25(19):10450. doi: 10.3390/ijms251910450 (PMC11477400; doi:10.3390/ijms251910450)
Supplement: Supplementary file 1 [file ijms-25-10450-s001.zip › ijms-3191160-supplementary.pdf]

Supplementary Table S1. Primers for PCR amplification

Names of primers	Primer sequences (5'-3')
StDAM5-F	gactcactatagggcgaattcAATGTTTGGTATTTAGTTAAAAGATAAGTTAG TT
StDAM5-R	gattcgcgaacgcgtgagctcATTGTAGGGTCATTCGGGGTG
StABF2-F	gactcactatagggcgaattcCCCTTCATACAAGGGCTAGTACTACA
StABF2-R	gattcgcgaacgcgtgagctcTTTTTTTAAAAAGAAGTCGGTAGCC
StGOLS2-F	gactcactatagggcgaattcGAATGGGCAGCATGGTCG
StGOLS2-R	gattcgcgaacgcgtgagctcTCAAATTAAAATTAACTACCCTACACGA
StLBD30-F	gactcactatagggcgaattcAAGTTCGACGAATGATATTTGACATT
StLBD30-R	gattcgcgaacgcgtgagctcAGGCTAAACCTACAAGATTTTGTGG
L-DAM5-pGreenII 0800-LUC-F	CCCCCTCGAGGTCGACAATGTTTGGTATTTAGTTAAAAGA
L-DAM5-pGreenII 0800-LUC-R	ACTAGTGGATCCCCCGGGATTGTAGGGTCATTCGGGGT
L-ABF2-pGreenII 0800-LUC-F	CCCCCTCGAGGTCGACCCCTTCATACAAGGGCTAGTAC
L-ABF2-pGreenII 0800-LUC-R	ACTAGTGGATCCCCCGGGTTTTTTTAAAAAGAAGTCGGTAGCC
L-GOLS2-pGreenII 0800-LUC-F	CCCCCTCGAGGTCGACGAATGGGCAGCATGGTCGC
L-GOLS2-pGreenII 0800-LUC-R	ACTAGTGGATCCCCCGGGTCAAATTAAAATTAACTACCC
L-LBD30-pGreenII 0800-LUC-F	CCCCCTCGAGGTCGACAAGTTCGACGAATGATATTTGAC
L-LBD30-pGreenII 0800-LUC-R	ACTAGTGGATCCCCCGGGAGGCTAAACCTACAAGATTTTGTGG

Supplementary Table S2. qRT-PCR specific primers

Names of primers	Primer sequences (5'-3')
qPCR-StDAM5-F	GAGGCGTGATTTGCATTCCA
qPCR-StDAM5-R	TGACACGGCTCAAACCAGTT
qPCR-StABF2-F	CCATCAACCACAGCAACAGC
qPCR-StABF2-R	TCCACCCATTAGAGCAGTGC
qPCR-StGOLS2-F	CTACATTGGACCGGGCCTAC
qPCR-StGOLS2-R	CAGGTGGGTAAACTGGCTCA
qPCR-StLBD30-F	CTGGAAAGCGTGTTTCAGGG
qPCR-StLBD30-R	CTCCAAACACCTTGTGCACG
EF1α-F	GATGGTCAGACCCGTGAACA
EF1α-R	CCTTGGAGTACTTCGGGGTG

Supplementary Table S3. Softwares of bioinformatics analysis

Tool	URL/Software
NCBI	https://www.ncbi.nlm.nih.gov/
ProtParam	https:// expasy.org/tools/protparam.html
Gene Structure Dispaly Server	http://gsds.gao-lab.org/
ProtScale	http://ca.expasy.org/tools/protscale.html
TMHMMServer v.2.0	http://www.cbs.dtu.dk/services/TMHMM/
MEGA7.0	https://www.megasoftware.net/
SOPMA	https://npsa-prabi.ibcp.fr/cgi-bin/npsa_automat.pl?page=npsa_sopma.html
SWISS-MODEL	http://swissmodel.expasy.org/
PlantCARE	https://bioinformatics.psb.ugent.be/webtools/plantcare/html/
SMART Main page	SMART: Sequence analysis results for M1BBZ0_SOLTU (embl.de)
SignalP	SignalP 4.1 - DTU Health Tech - Bioinformatic Services

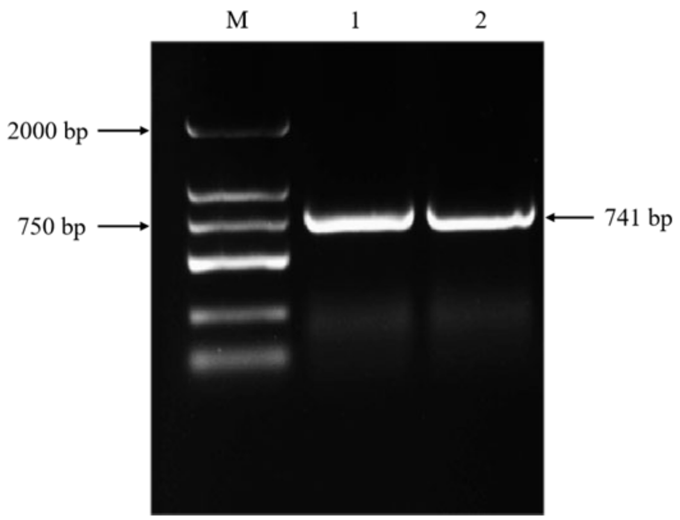

Supplementary Figure S1. Electrophoresis of PCR products of *StTCP7* gene in potato. M: DNA marker DL 2000; 1-2: The target fragment.

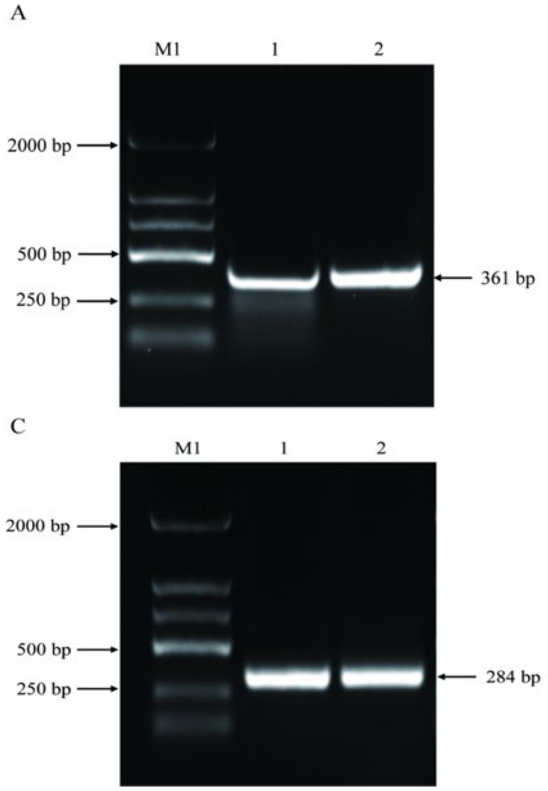

Supplementary Figure S2. Electrophoresis of PCR products in promoter region of target gene. M1: DNA Marker DL 2000; 1-2: promoter region of target gene. A: *StDAM5*; B: *StGOLS2*; C: *StABF2*; D: *StLBD30*.

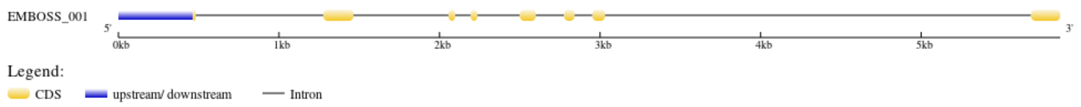

Supplementary Figure S3. Gene structure analysis of *StDAM5*. CDS: coding sequence; upstream/downstream: upstream/downstream; Intron: intron.

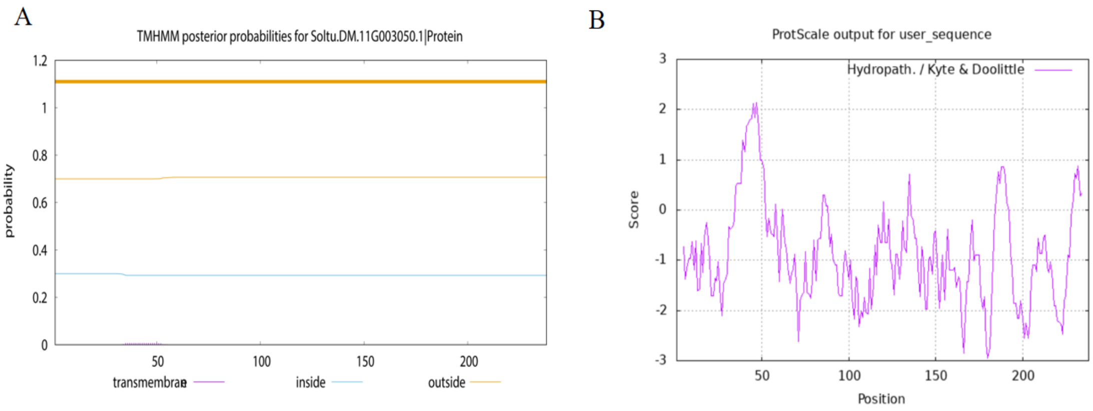

Supplementary Figure S4. A: Prediction of *StDAM5* amino acid transmembrane structure; B: Affinity/hydrophobicity analysis of *StDAM5* amino acid sequence.

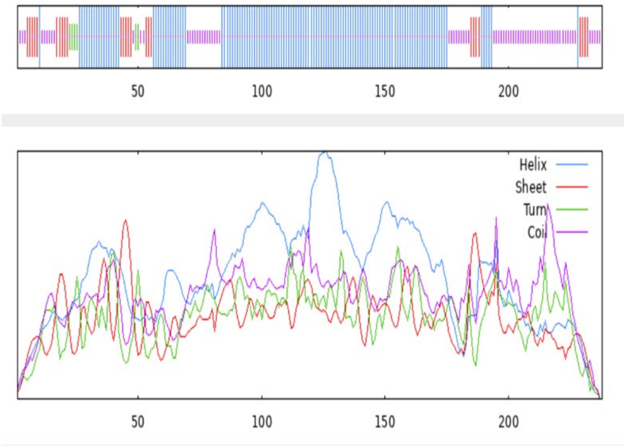

Supplementary Figure S5. Secondary structure of protein encoded by *StDAM5* gene.

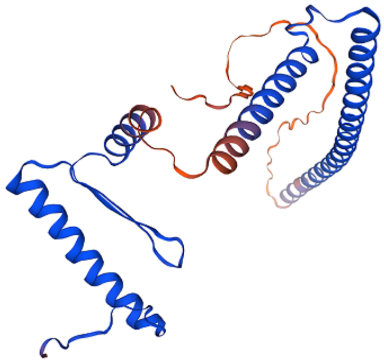

Supplementary Figure S6. The tertiary structure of protein encoded by *StDAM5* of potato.

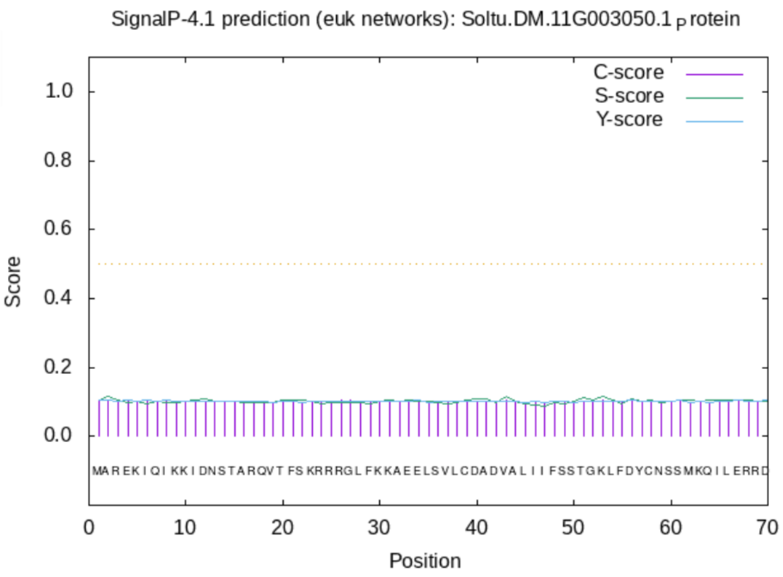

Supplementary Figure S7. Prediction of signal peptides.

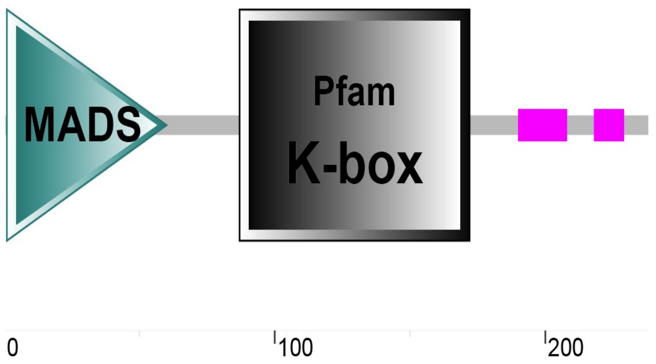

Supplementary Figure S8. Conservative structural domain prediction.

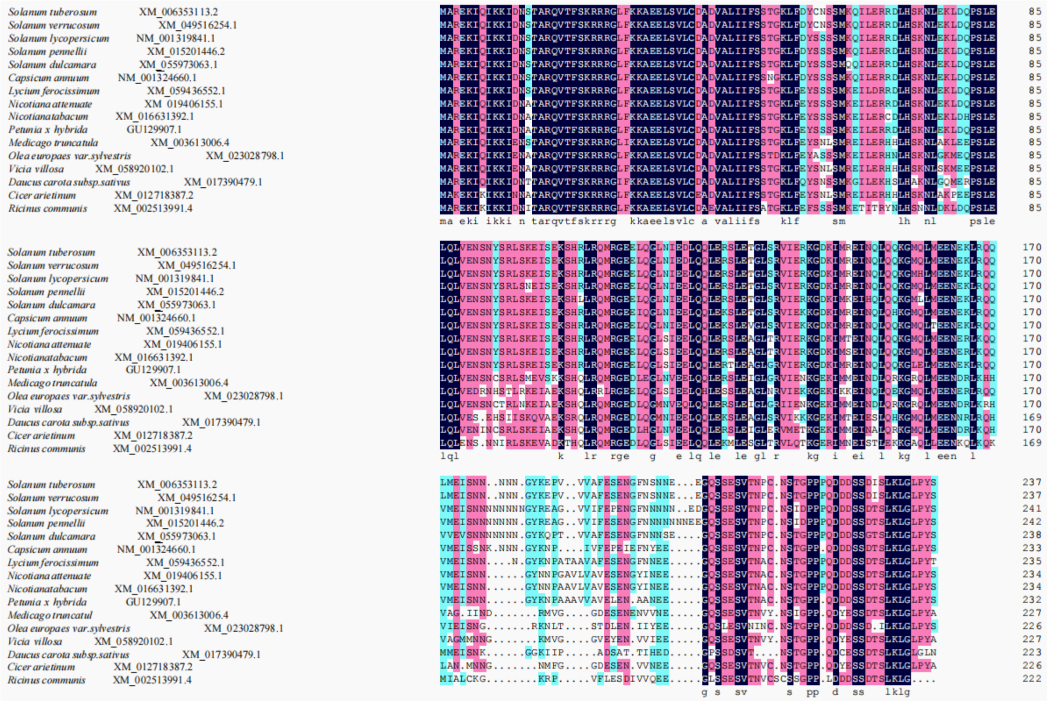

Supplementary Figure S9. Multiple comparisons between the amino acid sequence of StDAM5 and homologous sequences of other species. Black: identical sequences; Pink: more than 75 percent sequence similarity; Blue: more than 50 percent sequence similarity; White: less than 30 percent sequence similarity.

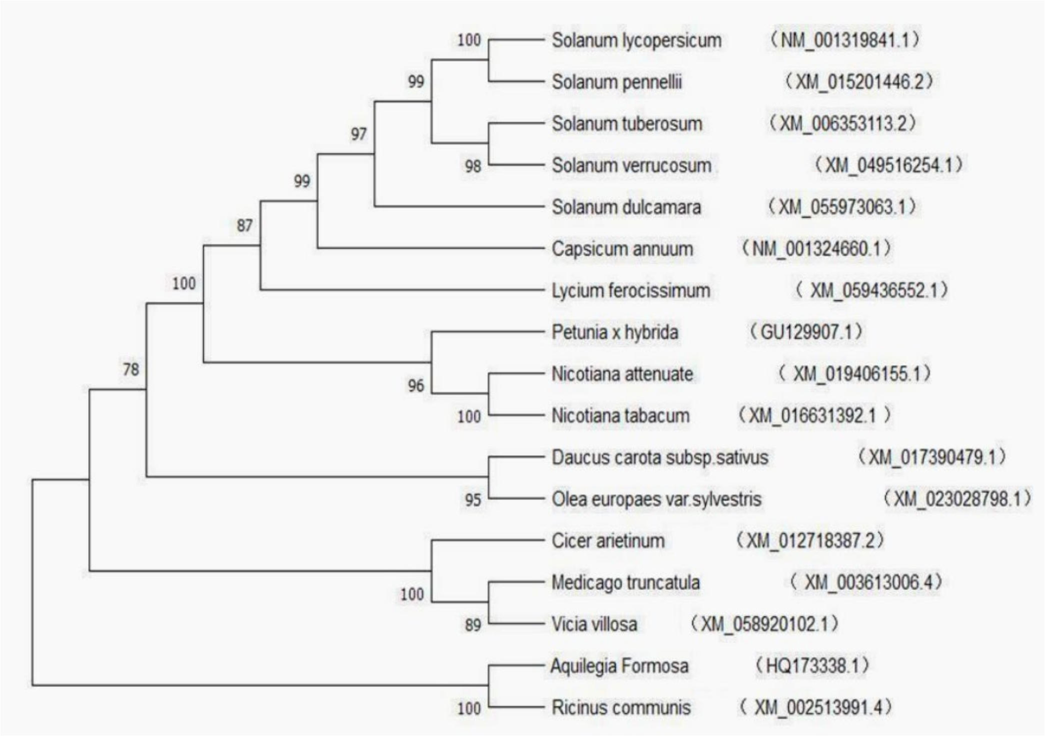

Supplementary Figure S10. Phylogenetic evolutionary tree analysis of the StDAM5 amino acid sequence and homologous.
